# Surgeon C. H. Wilcox on Sick Leave

**Published:** 1862-11

**Authors:** 


SURGEON C. H. WILCOX ON SICK LEAVE.—We are sorry to learn that Dr. Wilcox has been compelled to return home for the purpose of regaining his health. With his ripe experience he has served his country faithfully, indeed, with too untiring devotion since the commencement of the war, until at length, worn out with labor and fatigue, he has returned home where we hope soon to see him again restored to his former health and strength. As Surgeon of the 21st Reg't N. Y. S. V., and as acting Brigade Surgeon and Medical Director, he has been able to render the most valuable service; and perhaps we are not saying too much when we publish the opinion we have so often heard expressed by his medical associates in the army, "that no physician in the service enjoys more fully than Dr. Wilcox, the confidence and respect of the medical staff, officers and men."

